# Effects of Probiotic and Synbiotic Supplementation on Body Composition and Glucose Metabolism in Children and Adolescents with Overweight or Obesity: A Systematic Review and Meta-Analysis

**DOI:** 10.3390/nu18152539

**Published:** 2026-08-04

**Authors:** Ye Tao, Wei Wang, Ye Zhang, Mujuan Zhao, Zifu Xu, Haonan Yan, Feilong Zhu

**Affiliations:** 1 Department of Physical Education, Sejong University, Seoul 05006, Republic of Korea; ty231004@sju.ac.kr (Y.T.);; 2 Department of Sports Sociology, Kyungpook National University, Sangju Campus, Sangju 37224, Republic of Korea; 3 Department of Physical Education, Gangneung Campus, Kangwon National University, Gangneung 25457, Republic of Korea; 4 School of Sports Medicine and Rehabilitation, Beijing Sport University, Beijing 100084, China

**Keywords:** probiotics, synbiotics, children and adolescents, overweight, obesity, body composition, glucose metabolism

## Abstract

Background/Objectives: Childhood and adolescent overweight and obesity are associated with adverse body composition and impaired glucose metabolism. Probiotic and synbiotic supplementation may modulate the gut microbiota and has been proposed as a potential adjunctive strategy for obesity management. This systematic review and meta-analysis aimed to evaluate the effects of probiotic and synbiotic supplementation on body composition and glucose metabolism in children and adolescents with overweight or obesity. Methods: This review was conducted according to the PRISMA 2020 statement. PubMed, Embase, Web of Science, the Cochrane Library, and CNKI were searched from inception to 5 May 2026. Randomized controlled trials evaluating probiotic or synbiotic supplementation in children and adolescents with overweight or obesity were included. Outcomes included BMI z-score, BMI, waist circumference, body fat percentage, fasting glucose, fasting insulin, and HOMA-IR. Random-effects meta-analyses, sensitivity analyses, and subgroup analyses according to intervention type (probiotic versus synbiotic supplementation) were performed using mean differences (MDs) and 95% confidence intervals (CIs). Risk of bias was assessed using RoB 2, and certainty of evidence was evaluated using GRADE. Results: Sixteen randomized controlled trials were included. Probiotic or synbiotic supplementation did not significantly reduce BMI z-score (MD = −0.03, 95% CI −0.11 to 0.05), waist circumference (MD = 0.60 cm, 95% CI −1.57 to 2.77), or body fat percentage (MD = −0.29 percentage points, 95% CI −1.64 to 1.06). A modest reduction in BMI was observed (MD = −0.91 kg/m^2^, 95% CI −1.81 to −0.01), although this finding should be interpreted cautiously because no significant improvement was observed in BMI z-score. No significant effects were found for fasting glucose, fasting insulin, or HOMA-IR. Subgroup analyses showed no significant differences between probiotic and synbiotic supplementation for BMI, BMI z-score, or waist circumference, whereas a significant subgroup difference was observed for fasting glucose. Conclusions: Probiotic or synbiotic supplementation may be associated with a modest reduction in BMI; however, the certainty of this evidence was low, which limits confidence in its clinical implications. Current evidence does not support clear benefits for BMI z-score, waist circumference, body fat percentage, or glucose metabolism. The subgroup findings, particularly for fasting glucose, should be interpreted cautiously because they were based on a limited number of studies. Further high-quality randomized controlled trials are warranted.

## 1. Introduction

The prevalence of overweight and obesity among children and adolescents has increased substantially worldwide over recent decades, becoming a major public health concern affecting pediatric health. Global epidemiological evidence indicates that the burden of obesity in children and adolescents has increased markedly since the 1990s, and this trend is no longer confined to high-income countries but increasingly affects low- and middle-income countries and regions [1]. In children and adolescents, who are still undergoing growth and development, overweight and obesity are not only reflected by increased body mass index (BMI), but are also commonly accompanied by increased waist circumference, excessive body fat accumulation, and unfavorable body composition. These conditions may further impair cardiorespiratory fitness, physical performance, participation in physical activity, and overall physical development. In addition, childhood and adolescent obesity is closely associated with insulin resistance, impaired fasting glucose, and other abnormalities in glucose metabolism. Excess adiposity may also persist into adulthood, increasing the long-term risk of type 2 diabetes, cardiovascular disease, and other metabolic disorders [2,3,4,5]. Therefore, identifying effective strategies to improve body composition, glucose metabolism, and overall health in children and adolescents with overweight or obesity is of considerable clinical and public health importance.

Lifestyle intervention remains the cornerstone of overweight and obesity management in children and adolescents. Dietary modification, increased physical activity, reduced sedentary behavior, and regular exercise training are among the most commonly recommended strategies. For children and adolescents with overweight or obesity, exercise interventions are valuable not only for weight control but also for improving body composition, enhancing cardiorespiratory fitness, and promoting metabolic health. Previous studies have suggested that lifestyle interventions combining dietary modification and physical activity may reduce body fat and improve obesity-related health indicators in pediatric populations [6]. Exercise training may also improve glucose metabolism by enhancing skeletal muscle glucose uptake, increasing insulin sensitivity, and regulating energy metabolism, thereby potentially benefiting fasting insulin and insulin resistance [7,8]. However, in real-world settings, the effectiveness of lifestyle interventions is often limited by poor adherence, differences in family and school environments, limited intervention duration, and variability in individual responses. As a result, improvements in BMI, waist circumference, body fat percentage, or glucose metabolism may remain unsatisfactory in some children and adolescents. Therefore, it is important to explore safe, feasible, and potentially effective adjunctive strategies that may further improve body composition and glucose metabolism when used alongside lifestyle and exercise interventions.

In recent years, the role of the gut microbiota in obesity and related metabolic abnormalities has received increasing attention. The gut microbiota may participate in host fat accumulation, insulin sensitivity, and glucose homeostasis through multiple pathways, including the regulation of energy harvest and storage, short-chain fatty acid production, intestinal barrier function, low-grade inflammation, and bile acid metabolism [9,10]. Compared with individuals of normal weight, children and adolescents with overweight or obesity may exhibit reduced gut microbial diversity, altered microbial composition, and abnormal metabolic functions, which may be associated with unfavorable body composition and impaired glucose metabolism [11]. Consequently, modulation of the gut microbiota has been proposed as a potential approach for improving obesity-related metabolic health. Probiotics may improve the intestinal microecological environment by supplying beneficial live microorganisms, whereas synbiotics, which combine probiotics with prebiotics, may further promote the colonization and metabolic activity of beneficial bacteria. These mechanisms may potentially influence fat accumulation, insulin resistance, and glucose regulation [12,13].

Although probiotics and synbiotics have a plausible biological basis for modulating the gut microbiota and improving obesity-related metabolic abnormalities, their actual effects in children and adolescents with overweight or obesity remain uncertain. Previous randomized controlled trials have varied substantially in probiotic strains, intervention doses, supplementation duration, use of concurrent dietary or exercise guidance, and participant characteristics. As a result, findings regarding BMI, waist circumference, body fat percentage, fasting glucose, insulin, and homeostatic model assessment of insulin resistance (HOMA-IR) have been inconsistent across studies [14,15]. Existing systematic reviews and meta-analyses have often included probiotics, prebiotics, and synbiotics together and have evaluated a broad range of cardiometabolic outcomes, such as lipid profiles, inflammatory markers, and liver function indices [16,17]. However, from the perspective of pediatric health and obesity management, body composition and glucose metabolism are more direct indicators of obesity severity, physical health status, and metabolic risk. Therefore, a more focused evaluation of the effects of probiotic and synbiotic supplementation on body composition and glucose metabolism in children and adolescents with overweight or obesity is warranted to provide clearer and more clinically interpretable evidence.

Although probiotics and synbiotics differ in composition, both interventions target gut microbiota modulation and have been investigated as adjunctive microbiota-targeted strategies for pediatric obesity management. Given the limited number of randomized controlled trials in this population, probiotics and synbiotics were considered together in the primary analysis to provide a more comprehensive assessment of the effects of microbiota-targeted supplementation on pediatric obesity-related outcomes. Subgroup analyses were additionally performed to explore potential differences between probiotic and synbiotic supplementation. Based on this background, the present systematic review and meta-analysis aimed to synthesize evidence from randomized controlled trials evaluating the effects of probiotic and synbiotic supplementation on body composition and glucose metabolism in children and adolescents with overweight or obesity. The outcomes of interest included BMI z-score, BMI, waist circumference, body fat percentage, fasting glucose, fasting insulin, and HOMA-IR. This review was designed to provide targeted evidence for pediatric obesity management, health promotion, and the use of adjunctive nutritional interventions in this population.

## 2. Materials and Methods

### 2.1. Study Design

This systematic review and meta-analysis was conducted in accordance with the Preferred Reporting Items for Systematic Reviews and Meta-Analyses 2020 statement (PRISMA 2020) [18]. The completed PRISMA checklist is provided in Supplementary File S1. The study protocol was registered in the International Prospective Register of Systematic Reviews (PROSPERO; registration number: CRD420261395231). The eligibility criteria were developed according to the PICOS framework, including population, intervention, comparator, outcomes, and study design, to evaluate the effects of probiotic and synbiotic supplementation on body composition and glucose metabolism in children and adolescents with overweight or obesity [19].

### 2.2. Search Strategy

A systematic literature search was conducted in five electronic databases: PubMed, Embase, Web of Science, the Cochrane Library, and CNKI. The databases were searched from inception to 5 May 2026 to identify randomized controlled trials evaluating the effects of probiotic or synbiotic supplementation on body composition and glucose metabolism in children and adolescents with overweight or obesity. The initial search identified 1155 records, including 494 from PubMed, 350 from Web of Science, 146 from the Cochrane Library, 131 from Embase, and 34 from CNKI.

The search strategy combined controlled vocabulary terms and free-text terms related to children and adolescents, overweight or obesity, probiotics, synbiotics, body composition, glucose metabolism, and randomized controlled trials. The search strategy was adapted for each database according to its specific syntax and indexing system. No language restrictions were applied. In addition, the reference lists of included studies and relevant reviews were manually screened to identify additional potentially eligible studies. The complete search strategies for all databases are provided in Supplementary File S2. Grey literature, clinical trial registries, and preprint servers were not searched in this review.

### 2.3. Inclusion and Exclusion Criteria

The eligibility criteria were developed according to the PICOS framework. Studies were considered eligible if they met the criteria detailed in Table 1.

The exclusion criteria were as follows: (1) cross-sectional studies, cohort studies, case–control studies, reviews, meta-analyses, case reports, conference abstracts, animal studies, or cell experiments; (2) non-randomized controlled trials; (3) studies including participants with secondary obesity or known genetic, congenital, endocrine, or severe psychiatric disorders; (4) studies in which participants received anti-obesity medications during the study period or had undergone bariatric surgery; (5) studies in which the intervention consisted of prebiotic supplementation alone rather than probiotic or synbiotic supplementation; (6) studies that did not report the body composition or glucose metabolism outcomes of interest or did not provide data suitable for quantitative synthesis; (7) duplicate publications, overlapping datasets, or companion reports of the same study; and (8) crossover trials with clear carry-over effects and no available first-period data suitable for quantitative synthesis.

### 2.4. Study Selection

Study selection was conducted independently by two reviewers (Y.T. and F.Z). First, duplicate records were removed using EndNote X9 (Clarivate Analytics, Philadelphia, PA, USA). The remaining records were then screened by title and abstract to exclude studies that were clearly irrelevant to the study design, population, intervention, comparator, or outcomes of interest. For records that potentially met the eligibility criteria or could not be clearly judged based on the title and abstract, the full texts were retrieved for further assessment.

Subsequently, the two reviewers independently assessed the full-text articles according to the predefined inclusion and exclusion criteria. Any disagreements during the screening process were resolved through discussion. If consensus could not be reached, a third reviewer (W.W.) was consulted for final judgment. The study selection process was recorded and presented using a PRISMA 2020 flow diagram.

### 2.5. Data Extraction

Data were independently extracted from the included studies by two reviewers (Y.T. and F.Z.) and checked by a third reviewer (W.W.). The extracted information mainly included the first author; publication year; country or region; study design; sample size; participants’ age; intervention; comparator; type of probiotic or synbiotic; strain composition; dosage; form of supplementation; intervention duration; whether dietary, exercise, or lifestyle guidance was provided; and outcomes of interest.

The outcomes extracted in this review included BMI z-score, BMI, waist circumference, body fat percentage, fasting glucose, fasting insulin, and HOMA-IR. For continuous outcomes, endpoint means, standard deviations, and sample sizes were preferentially extracted. When multiple intervention time points were reported, data at the end of the intervention were extracted. When continuous data were reported as medians with interquartile ranges, means and standard deviations were estimated using previously recommended methods. For data that were not directly available, values were extracted from tables, figures, or Supplementary Materials. If the relevant data were still unavailable, the outcome was not included in the quantitative synthesis and was described narratively when appropriate.

For multi-arm studies, only study arms that met the PICOS criteria and allowed a clear comparison between probiotic or synbiotic supplementation and the control intervention were extracted in order to avoid double-counting participants or introducing additional differences due to co-interventions. Any disagreements during data extraction were resolved through discussion between Y.T. and F.Z. When necessary, a third reviewer W.W. was consulted for final judgment.

### 2.6. Risk-of-Bias Assessment

Two reviewers (Y.T. and F.Z.) independently assessed the risk of bias of the included studies using the revised Cochrane risk-of-bias tool for randomized trials, version 2 (RoB 2). The assessment covered five domains: bias arising from the randomization process, bias due to deviations from intended interventions, bias due to missing outcome data, bias in measurement of the outcome, and bias in selection of the reported result. The risk of bias for each domain was judged as “low risk”, “some concerns”, or “high risk”, and an overall risk-of-bias judgment was then assigned for each study. Any disagreements between the two reviewers were resolved through discussion. Inter-rater agreement was not formally quantified using statistical measures such as Cohen’s kappa, as disagreements were resolved through consensus discussion after independent assessment. If consensus could not be reached, a third reviewer (W.W.) was consulted for final judgment.

### 2.7. Statistical Analysis

Data synthesis was performed using Review Manager 5.4.1. Meta-analysis data were independently entered and checked by two reviewers (Z.X. and Y.Z.) to ensure accuracy. A meta-analysis was conducted when at least two studies reported the same outcome and the effect measures were comparable. When quantitative pooling was inappropriate because of differences in study design, data format, reporting method, or measurement scale, the findings were summarized narratively.

The outcomes analyzed in this review included body composition and glucose metabolism outcomes. Body composition outcomes included BMI z-score, BMI, waist circumference, and body fat percentage. Glucose metabolism outcomes included fasting glucose, fasting insulin, and HOMA-IR. As all outcomes included in the quantitative synthesis were continuous variables, and the same outcomes were measured using identical or convertible units across studies, mean differences (MDs) with 95% confidence intervals (CIs) were used as the pooled effect estimates. Unit conversions were performed before pooling when necessary to ensure comparability across studies. Although variability existed in assessment methods and participant characteristics across studies, MDs were selected because the included outcomes were reported using identical or clinically interpretable units, allowing direct interpretation of the magnitude of intervention effects. SMDs were not used because standardization could potentially reduce the clinical interpretability of absolute changes in outcomes such as BMI, fasting glucose, and HOMA-IR.

For the primary meta-analysis, endpoint values at the end of the intervention were preferentially used. Studies that reported only changes from baseline, percentage changes, or model-based estimates and that could not be reasonably combined with endpoint means and standard deviations were not included in the primary quantitative analysis but were described narratively when appropriate. When continuous outcomes were reported as medians with interquartile ranges, means and standard deviations were estimated using previously recommended methods before inclusion in the analysis.

Considering the potential clinical and methodological heterogeneity among the included studies in terms of probiotic or synbiotic type, strain composition, dosage, intervention duration, participant characteristics, baseline metabolic status, and whether dietary, exercise, or lifestyle guidance was provided, all meta-analyses were performed using the inverse-variance method with a random-effects model. Between-study heterogeneity was assessed using Cochran’s Q test and the *I*^2^ statistic. *I*^2^ values of approximately 25%, 50%, and 75% were considered to indicate low, moderate, and high heterogeneity, respectively. A two-sided *p* value < 0.05 was considered statistically significant.

To assess the robustness of the findings, leave-one-study-out sensitivity analyses were performed for outcomes included in the meta-analysis. Each study was sequentially removed, and the pooled effect estimates were recalculated to evaluate whether the overall findings were influenced by any individual study. The robustness of the results was assessed by examining changes in the direction, magnitude, and statistical significance of pooled estimates after removal of individual studies. Additional influence analyses beyond the leave-one-study-out approach were not performed because the number of studies available for several outcomes was limited, which may reduce the reliability and interpretability of more complex influence diagnostics. Subgroup analyses were only performed according to intervention type because the number of studies was insufficient to support additional subgroup analyses based on probiotic strain, dosage, intervention duration, obesity phenotype, baseline metabolic status, or lifestyle co-interventions. Statistical tests for publication bias, such as Egger’s regression test, were not performed because the number of studies included for most outcomes was limited, and these tests may have insufficient statistical power when fewer studies are available. Therefore, the results of publication bias assessment should be interpreted cautiously.

### 2.8. Certainty of Evidence Assessment

Two reviewers (Z.X. and H.Y.) assessed the certainty of evidence for each primary outcome using the Grading of Recommendations Assessment, Development and Evaluation (GRADE) approach. Because all studies included in the quantitative analyses were randomized controlled trials, the initial certainty of evidence was rated as high. The certainty of evidence was then evaluated across five domains: risk of bias, inconsistency, indirectness, imprecision, and publication bias. When serious or very serious concerns were identified in any domain, the certainty of evidence was downgraded accordingly.

The certainty of evidence was classified into four levels: high, moderate, low, and very low. In this review, the certainty of evidence was assessed separately for seven outcomes: BMI z-score, BMI, waist circumference, body fat percentage, fasting glucose, fasting insulin, and HOMA-IR. A summary of findings table was prepared to present the pooled effect estimates, certainty of evidence, and the main GRADE domains responsible for downgrading the certainty for each outcome. Any disagreements between the two reviewers during certainty assessment were resolved through discussion. When necessary, a third reviewer (M.Z.) was consulted for final judgment. The results are presented in Table 2.

## 3. Results

### 3.1. Literature Search

**Figure 1:**
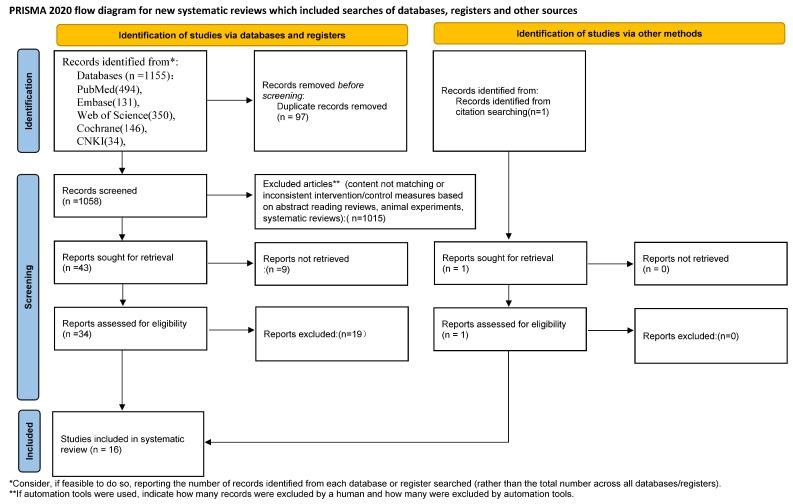
PRISMA 2020 flow diagram of study selection. Source: Page MJ, et al. [18]. This work is licensed under CC BY 4.0. To view a copy of this license, visit https://creativecommons.org/licenses/by/4.0 (accessed on 31 July 2026).

The initial database search identified 1155 records, including 494 from PubMed, 131 from Embase, 350 from Web of Science, 146 from the Cochrane Library, and 34 from CNKI. After removing 97 duplicate records, 1058 records were screened by title and abstract. Of these, 1015 records were excluded during the initial screening, and 43 reports were sought for full-text retrieval. Nine reports could not be retrieved, leaving 34 reports for full-text assessment. In addition, one potentially relevant report was identified through citation searching and assessed in full text. Therefore, a total of 35 full-text reports were assessed for eligibility, of which 19 were excluded because they did not meet the inclusion criteria. Finally, 16 randomized controlled trials were included in the systematic review. The study selection process is shown in Figure 1.

### 3.2. Study Characteristics

The basic characteristics of the included studies are presented in Table 3. A total of 16 randomized controlled trials were included in this systematic review and meta-analysis [14,15,20,21,22,23,24,25,26,27,28,29,30,31,32,33]. The included studies were conducted in Italy [20,29], Taiwan, China [21], Iran [14,22,27,31], Denmark [23], Turkey [24,26], the United States [30], Thailand [15], Sri Lanka [25], Spain [28], and mainland China [32,33]. The participants were children and adolescents with overweight or obesity, with an approximate age range of 5 to 18 years. Several studies specifically included participants with obesity-related conditions, such as non-alcoholic fatty liver disease, insulin resistance, or severe obesity. Baseline BMI status varied across studies, with reported BMI values or BMI z-scores indicating differences in obesity characteristics among participants. The sample sizes ranged from 8 completers to 105 randomized participants, and the intervention duration ranged from 4 to 24 weeks.

All included studies were randomized controlled trials with parallel-group designs. Most studies used double-blind or triple-blind designs, whereas several studies were open-label or pilot trials. The interventions included single-strain probiotics, multi-strain probiotics, and synbiotic formulations, with substantial variation in strain composition, formulation type, dosage, and intervention duration across studies. In addition to probiotic or synbiotic supplementation, some studies incorporated dietary counselling, physical activity recommendations, or structured lifestyle interventions, whereas others evaluated supplementation without additional behavioural interventions.

The outcomes reported in the included studies mainly included BMI z-score, BMI, waist circumference, body fat percentage, fasting glucose, fasting insulin, and HOMA-IR. The number of studies included in the quantitative synthesis varied across outcomes. Among body composition outcomes, BMI z-score, BMI, and waist circumference were the most frequently reported outcomes. Among glucose metabolism outcomes, fasting glucose, fasting insulin, and HOMA-IR were the main indicators assessed. Detailed information regarding probiotic and synbiotic formulations, including strain/product characteristics, dosage, intervention duration, population characteristics, and individual study-level outcome findings, is provided in Supplementary Table S1.

### 3.3. Risk-of-Bias Assessment

**Figure 2:**
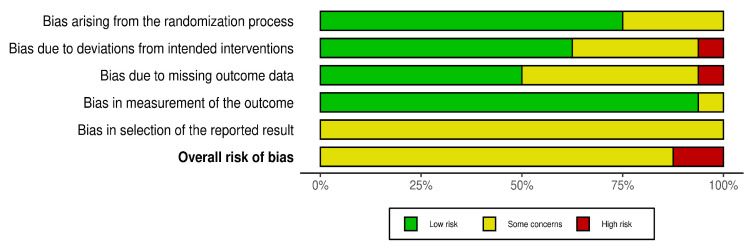
Risk-of-bias summary for the included randomized controlled trials.

**Figure 3:**
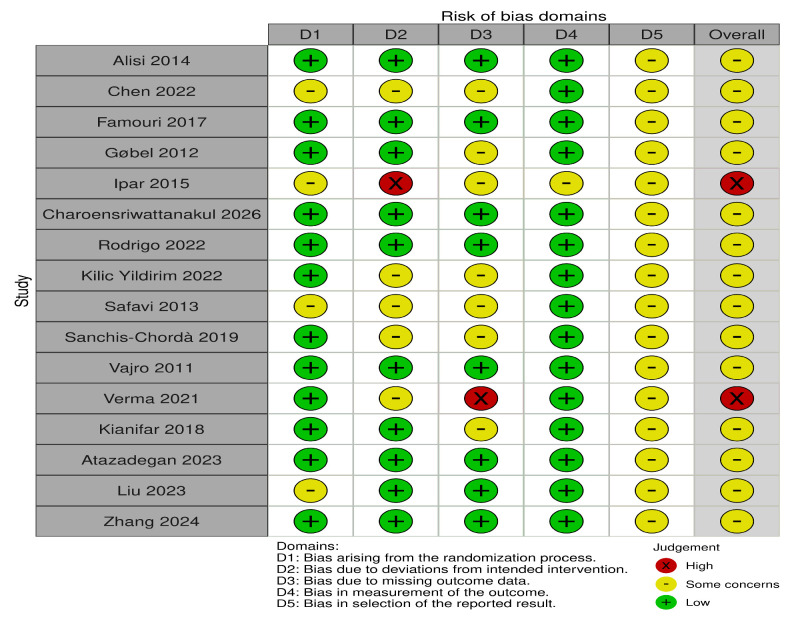
Risk-of-bias traffic-light plot for the included randomized controlled trials [14,15,20,21,22,23,24,25,26,27,28,29,30,31,32,33].

The results of the Cochrane RoB 2 assessment are shown in Figure 2 and Figure 3. Overall, most included studies were judged to have some concerns, and a small number were judged to have a high overall risk of bias. The main sources of concern were insufficient reporting of random sequence generation or allocation concealment; incomplete information on blinding, intervention adherence, co-interventions, and intention-to-treat analyses; and missing outcome data in some studies.

Most outcomes were measured using objective or standardized methods; therefore, the risk of bias in outcome measurement was generally low. However, selective reporting remained a concern in many studies because publicly available protocols or prespecified statistical analysis plans were often unavailable. These methodological limitations should be considered when interpreting the pooled effect estimates.

### 3.4. Meta-Analysis

#### 3.4.1. Effects on Body Composition Outcomes

**Figure 4:**
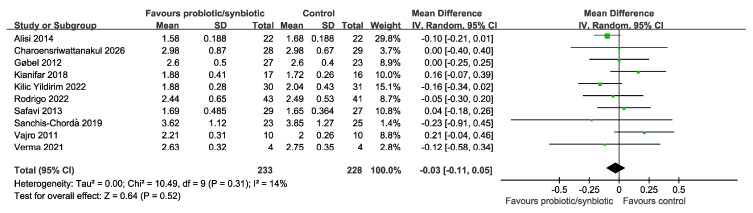
Forest plot of BMI z-score [15,20,23,25,26,27,28,29,30,31].

**Figure 5:**
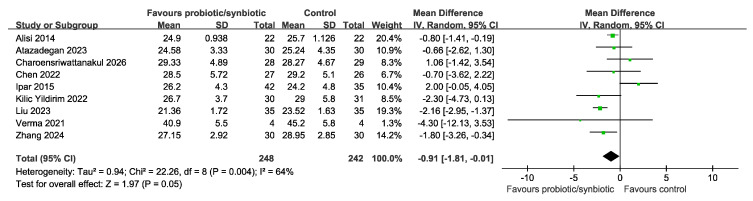
Forest plot of BMI [14,15,20,21,24,26,30,32,33].

**Figure 6:**
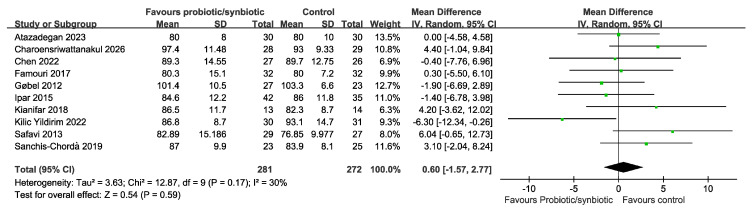
Forest plot of waist circumference [14,15,21,22,23,24,26,27,28,31].

**Figure 7:**
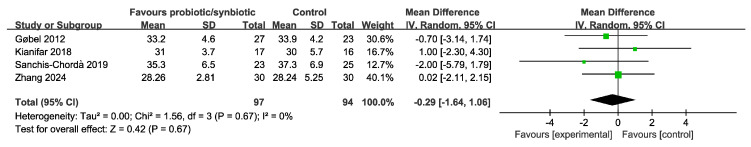
Forest plot of body fat percentage [23,28,31,32].

The effects of probiotic or synbiotic supplementation on body composition outcomes are presented in Figure 4, Figure 5, Figure 6 and Figure 7. Ten studies reported BMI z-score or BMI SDS [15,20,23,25,26,27,28,29,30,31]. The pooled analysis showed no significant reduction in BMI z-score compared with the control group (MD = −0.03, 95% CI −0.11 to 0.05; *I*^2^ = 14%) (Figure 4). Nine studies reported BMI [14,15,20,21,24,26,30,31,33], and probiotic or synbiotic supplementation was associated with a modest reduction in BMI (MD = −0.91 kg/m^2^, 95% CI −1.81 to −0.01; *I*^2^ = 64%) (Figure 5). Ten studies reported waist circumference [14,15,21,22,23,24,26,27,28,31], and the pooled result showed no statistically significant difference between the intervention and control groups (MD = 0.60 cm, 95% CI −1.57 to 2.77; *I*^2^ = 30%) (Figure 6). Four studies reported body fat percentage [23,28,31,33], and no significant effect was observed (MD = −0.29 percentage points, 95% CI −1.64 to 1.06; *I*^2^ = 0%) (Figure 7). Overall, the current evidence suggests that probiotic or synbiotic supplementation may be associated with a modest reduction in BMI; however, no significant improvements were observed in BMI z-score, waist circumference, or body fat percentage.

**Figure 8:**
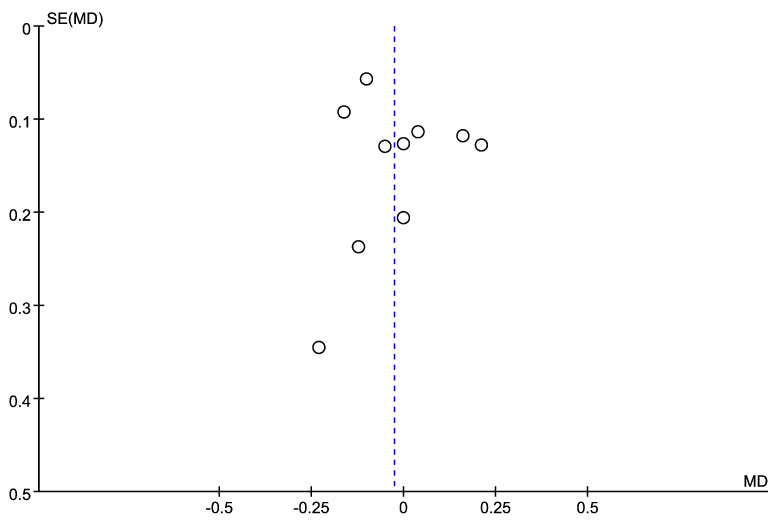
Funnel plot of BMI z-score.

**Figure 9:**
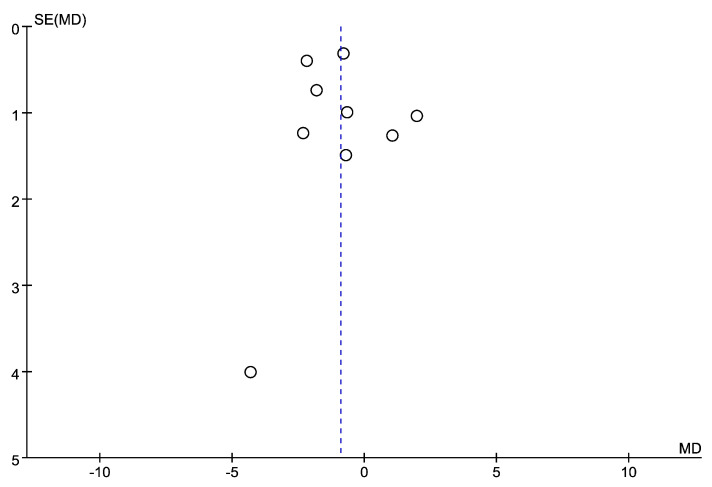
Funnel plot of BMI.

The funnel plots for body composition outcomes are shown in Figure 8 and Figure 9. Visual inspection did not reveal obvious asymmetry for BMI z-score, BMI, waist circumference, or body fat percentage. Nevertheless, because the number of included studies was relatively small, particularly for body fat percentage, the possibility of publication bias or small-study effects cannot be completely excluded.

Leave-one-study-out sensitivity analyses were performed for body composition outcomes by sequentially removing each individual study to assess the robustness of the pooled estimates. The detailed results are presented in Supplementary Tables S2 and S3. The pooled estimates remained consistent in direction, magnitude, and statistical significance after exclusion of individual studies, indicating that no single study substantially influenced the overall effect estimates. These findings suggest that the conclusions regarding body composition outcomes were robust. However, the observed reduction in BMI should be interpreted with caution because moderate heterogeneity remained and no corresponding improvement was observed for BMI z-score.

#### 3.4.2. Effects on Glucose Metabolism Outcomes

**Figure 10:**
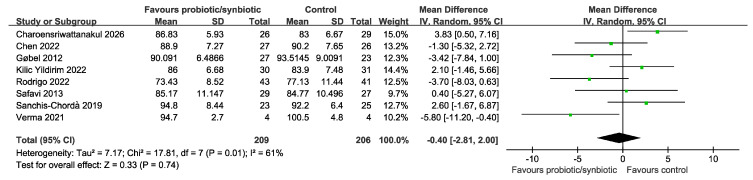
Forest plot of fasting glucose [15,21,23,25,26,27,28,30].

**Figure 11:**
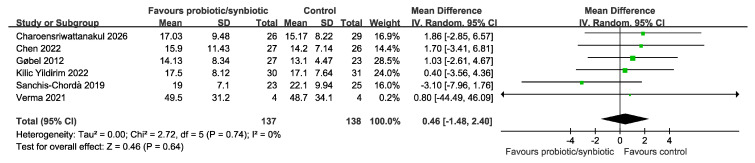
Forest plot of fasting insulin [15,21,23,26,28,30].

**Figure 12:**
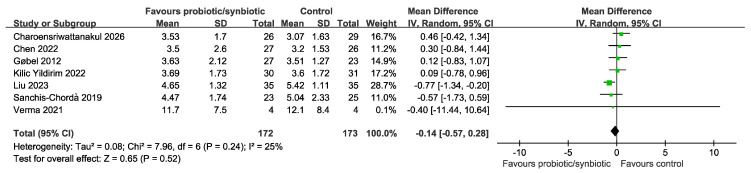
Forest plot of HOMA-IR [15,21,23,26,28,30,33].

The effects of probiotic or synbiotic supplementation on glucose metabolism outcomes are presented in Figure 10, Figure 11 and Figure 12. Eight studies reported fasting glucose [15,21,23,25,26,27,28,30]. The pooled analysis showed that probiotic or synbiotic supplementation did not significantly affect fasting glucose compared with the control group (MD = −0.40 mg/dL, 95% CI −2.81 to 2.00; *I*^2^ = 61%) (Figure 10). Six studies reported fasting insulin [15,21,23,26,28,30], and no statistically significant difference was observed between the intervention and control groups (MD = 0.46 μIU/mL, 95% CI −1.48 to 2.40; *I*^2^ = 0%) (Figure 11). Seven studies reported HOMA-IR [15,21,23,26,28,30,32], and the pooled analysis likewise demonstrated no significant difference between the two groups (MD = −0.14, 95% CI −0.57 to 0.28; *I*^2^ = 25%) (Figure 12).

Overall, the available evidence did not demonstrate significant improvements in fasting glucose, fasting insulin, or insulin resistance following probiotic or synbiotic supplementation in children and adolescents with overweight or obesity.

Leave-one-study-out sensitivity analyses were performed for glucose metabolism outcomes by sequentially removing each individual study to assess the robustness of the pooled estimates. The detailed results are presented in Supplementary Tables S4–S6. The pooled estimates remained consistent in direction, magnitude, and statistical significance after exclusion of individual studies, indicating that no single study substantially influenced the overall effect estimates. These findings suggest that the conclusions regarding glucose metabolism outcomes were robust, with no substantial changes in the direction, magnitude, or statistical significance of the pooled estimates.

#### 3.4.3. Subgroup Analysis According to Intervention Type

**Figure 13:**
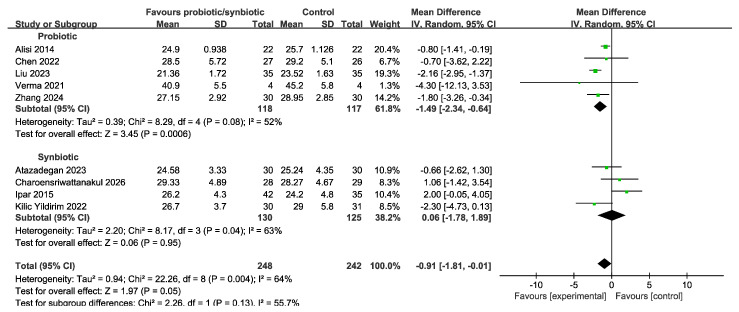
Subgroup analysis of BMI according to intervention type (1) [14,15,20,21,24,26,30,32,33].

**Figure 14:**
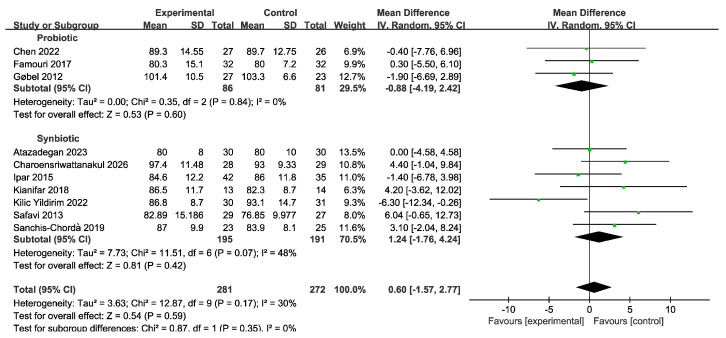
Subgroup analysis of BMI z-score according to intervention type [15,20,21,22,23,25,26,27,28,29,30,31].

**Figure 15:**
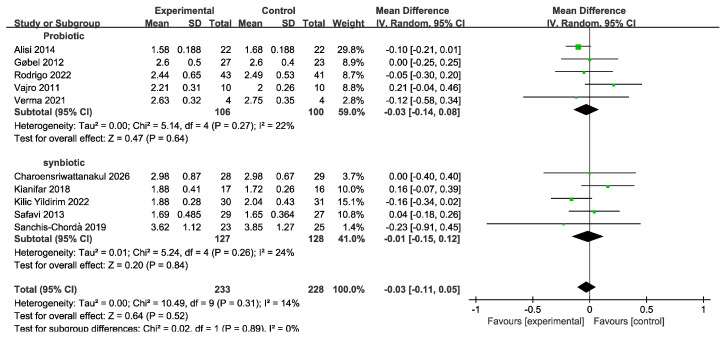
Subgroup analysis of waist circumference according to intervention type [15,23,26,27,28,31].

**Figure 16:**
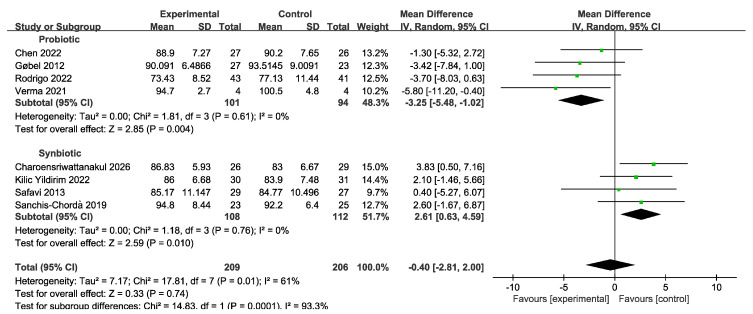
Subgroup analysis of fasting glucose according to intervention type [15,21,23,26,28,30].

Subgroup analyses according to intervention type (probiotic versus synbiotic supplementation) were performed for BMI, BMI z-score, waist circumference, and fasting glucose (Figure 13, Figure 14, Figure 15 and Figure 16). For BMI, probiotic supplementation was associated with a significant reduction compared with the control group (MD = −1.49 kg/m^2^, 95% CI −2.34 to −0.64), whereas synbiotic supplementation showed no significant effect (MD = 0.06 kg/m^2^, 95% CI −1.78 to 1.89). However, the test for subgroup differences was not statistically significant (χ^2^ = 2.26, *df* = 1, *p* = 0.13), indicating that the intervention type did not significantly modify the overall effect on BMI (Figure 13).

For BMI z-score, neither probiotic supplementation (MD = −0.03, 95% CI −0.14 to 0.08) nor synbiotic supplementation (MD = −0.01, 95% CI −0.15 to 0.12) showed a significant effect compared with the control group. Likewise, no significant subgroup difference was observed between probiotic and synbiotic supplementation (χ^2^ = 0.02, *df* = 1, *p* = 0.89) (Figure 14).

Similarly, subgroup analysis of waist circumference demonstrated no significant differences between probiotic and synbiotic supplementation (χ^2^ = 0.87, *df* = 1, *p* = 0.35), and neither subgroup showed a statistically significant reduction in waist circumference (Figure 15).

For fasting glucose, probiotic supplementation was associated with a reduction in fasting glucose (MD = −3.25 mg/dL, 95% CI −5.48 to −1.02), whereas synbiotic supplementation showed an increase in fasting glucose (MD = 2.61 mg/dL, 95% CI 0.63 to 4.59). The test for subgroup differences was statistically significant (χ^2^ = 14.83, *df* = 1, *p* = 0.0001), suggesting that intervention type may influence the effect on fasting glucose (Figure 16). However, this finding should be interpreted cautiously because the subgroup analyses were based on a limited number of studies and should be considered exploratory.

## 4. Discussion

This systematic review and meta-analysis included 16 randomized controlled trials and evaluated the effects of probiotic and synbiotic supplementation on body composition and glucose metabolism in children and adolescents with overweight or obesity. The results showed that probiotic or synbiotic supplementation was associated with a small reduction in BMI. However, no significant improvements were observed in BMI z-score, waist circumference, body fat percentage, fasting glucose, fasting insulin, or HOMA-IR. Sensitivity analyses showed that, after excluding Sensitivity analyses using sequential exclusion of individual studies showed that the direction, magnitude, and statistical significance of the pooled effects for glucose metabolism outcomes remained stable. For body composition outcomes, BMI z-score remained non-significant, while the reduction in BMI persisted, although moderate heterogeneity remained. These findings suggest that probiotics and synbiotics may have a limited effect on BMI, but their effects on more clinically stable indicators of adiposity and glucose metabolism remain uncertain.

The gut microbiota has received increasing attention in the development and progression of childhood and adolescent obesity. Previous studies have suggested that children and adolescents with obesity may have reduced gut microbial diversity, altered microbial composition, abnormal short-chain fatty acid metabolism, impaired intestinal barrier function, and low-grade inflammation, all of which may contribute to energy metabolism, fat accumulation, and insulin resistance [11,34]. Therefore, modulation of the gut microbiota through probiotics, prebiotics, or synbiotics has been proposed as a potential adjunctive strategy for pediatric obesity management [35,36]. However, clinical findings remain inconsistent. Differences in probiotic strains, strain combinations, dosage, intervention duration, background lifestyle interventions, and baseline metabolic status may all influence the observed effects of probiotic or synbiotic supplementation [37,38,39,40]. The present findings are generally consistent with this evidence base, suggesting that probiotic or synbiotic supplementation may have a modest effect on BMI, but clear and stable benefits for BMI z-score, body fat percentage, and glucose metabolism have not yet been demonstrated. Nevertheless, the substantial clinical heterogeneity among the included trials should be considered when interpreting these pooled findings. Differences in probiotic/synbiotic formulations, bacterial strains, dosage, intervention duration, baseline metabolic phenotypes, and concomitant lifestyle interventions may have contributed to variations in treatment effects. Therefore, the pooled estimates should be interpreted as average effects across heterogeneous interventions rather than evidence supporting a uniform benefit of all probiotic or synbiotic preparations in children and adolescents with overweight or obesity.

The small reduction in BMI observed in this review should be interpreted cautiously. Children and adolescents are in a period of growth and development, and BMI is influenced not only by adiposity but also by age, sex, height growth, and pubertal status. Compared with crude BMI, BMI z-score or BMI SDS better accounts for age- and sex-related growth differences and is therefore generally more informative in pediatric obesity research. In the present analysis, BMI showed a small reduction, whereas BMI z-score did not change significantly. This discrepancy suggests that probiotic or synbiotic supplementation may have only a limited effect on true obesity severity in children and adolescents. Moreover, although the pooled reduction of approximately 0.9 kg/m^2^ reached statistical significance, the clinical relevance of this magnitude of change remains uncertain, particularly regarding whether it can translate into meaningful long-term improvements in pediatric health, given the absence of consistent improvements in BMI z-score, waist circumference, body fat percentage, or metabolic outcomes. Duan et al. also reported that probiotic intervention reduced BMI but had no significant effects on waist circumference, insulin, or HOMA-IR [16]. Zhang et al., in a Bayesian network meta-analysis, suggested that different microbiota-targeted interventions may have different effects, although the certainty and consistency of evidence varied across outcomes [17]. Recent GRADE-based systematic reviews have similarly indicated that the certainty of evidence for probiotics or synbiotics in pediatric obesity is often low to moderate because of small sample sizes, heterogeneity, and risk of bias [39,40]. This discrepancy suggests that the observed BMI reduction may not necessarily represent a clinically meaningful improvement in obesity severity among children and adolescents. Therefore, although probiotic or synbiotic supplementation may have a modest effect on BMI, the absence of improvement in BMI z-score indicates that the clinical relevance of this finding remains uncertain.

The lack of significant changes in waist circumference and body fat percentage further suggests that probiotic or synbiotic supplementation may not have stable effects on central adiposity or body composition. Waist circumference reflects abdominal fat accumulation and central obesity risk, whereas body fat percentage more directly reflects adiposity than BMI. In this review, neither outcome showed significant improvement. This may partly be explained by the relatively short intervention duration in the included studies, which generally ranged from 4 to 24 weeks. Such a duration may be insufficient to produce measurable changes in fat distribution or body composition, particularly without sustained dietary control and regular physical activity. Previous reviews of nutritional adjunctive interventions in pediatric obesity have also emphasized that short-term supplementation alone is unlikely to produce consistent improvements in body composition without adequate lifestyle modification [41]. Therefore, probiotics and synbiotics should be regarded as potential adjuncts to lifestyle intervention rather than independent weight-loss treatments.

Regarding glucose metabolism, this review found no significant effects of probiotic or synbiotic supplementation on fasting glucose, fasting insulin, or HOMA-IR. Considering the certainty of evidence, the lack of significant effects on fasting glucose, fasting insulin, and HOMA-IR should be interpreted cautiously, although these outcomes were supported by moderate-certainty evidence according to the GRADE assessment. The results remained materially unchanged after sequentially excluding each individual study, suggesting that the findings for glucose metabolism outcomes were relatively robust. Several factors may explain these non-significant findings. First, although participants had overweight or obesity, fasting glucose may have been normal or near normal at baseline in some studies, leaving limited room for improvement. Second, improvements in glucose metabolism may require longer-term changes in body weight, adiposity, inflammation, and insulin sensitivity, whereas most included trials had relatively short intervention periods. Third, the included populations were heterogeneous, including children and adolescents with simple obesity, non-alcoholic fatty liver disease, insulin resistance, or severe obesity, and these subgroups may respond differently to probiotic or synbiotic supplementation. Previous reviews in adults or metabolically abnormal populations have suggested that probiotics or synbiotics may improve some insulin resistance indicators, but their effects on fasting glucose remain inconsistent [42,43,44]. Therefore, current evidence is insufficient to support probiotics or synbiotics as established interventions for improving insulin resistance or glycemic control in children and adolescents with overweight or obesity.

Subgroup analyses according to intervention type provided additional insights into the pooled findings. For BMI, probiotic supplementation showed a significant reduction, whereas synbiotic supplementation did not; however, the test for subgroup differences was not statistically significant. Likewise, no significant subgroup differences were observed for BMI z-score or waist circumference. In contrast, a statistically significant subgroup difference was observed for fasting glucose. Nevertheless, this finding was based on a limited number of studies within each subgroup and should therefore be considered exploratory rather than confirmatory. Whether probiotic and synbiotic interventions differ in their effects on fasting glucose requires further evaluation in adequately powered trials.

From a clinical and public health perspective, the management of childhood and adolescent overweight and obesity should continue to be based on dietary modification, regular physical activity, reduced sedentary behavior, behavioral management, and family- and school-based support. Probiotics and synbiotics are relatively easy to administer and are generally considered acceptable as adjunctive nutritional interventions. However, the present findings suggest that their actual effects may be limited, especially for glucose metabolism. Therefore, probiotic or synbiotic supplementation should not be recommended as a primary strategy for weight loss or improvement of insulin resistance. In selected children and adolescents with overweight or obesity, these supplements may be considered as adjuncts to lifestyle intervention, taking into account the specific strain evidence, product quality, safety, adherence, and individual metabolic status.

This review has several strengths. First, it focused on children and adolescents with overweight or obesity and analyzed two clinically relevant outcome domains: body composition and glucose metabolism. Second, probiotic and synbiotic interventions were included, while prebiotic-only studies were excluded, making the research question more focused. Third, the search included commonly used international databases and CNKI, improving the coverage of available evidence. In addition, risk of bias was assessed using the RoB 2 tool, and the certainty of evidence was evaluated using the GRADE approach, which helped provide a more transparent interpretation of the pooled findings.

Several limitations should also be acknowledged. First, the number of included studies and participants remained limited, particularly for body fat percentage, fasting insulin, and HOMA-IR, which may have reduced statistical power. In addition, endpoint values were primarily analyzed because change scores and adjusted estimates were inconsistently reported among the included studies. Although this approach facilitated quantitative synthesis and allowed inclusion of more eligible trials, it may have introduced potential bias because baseline differences between groups could not be fully accounted for. Second, considerable clinical heterogeneity existed among the included trials regarding probiotic or synbiotic formulations, probiotic strains, strain combinations, dosages, supplementation forms, intervention durations, baseline metabolic characteristics, and concomitant lifestyle interventions. Although random-effects models were applied to account for statistical heterogeneity, substantial clinical heterogeneity among the included trials, including differences in probiotic/synbiotic formulations, strains, dosages, intervention durations, participant characteristics, metabolic phenotypes, and co-interventions, may limit the interpretation and generalisability of the pooled estimates. Therefore, these pooled estimates should be interpreted as average effects across diverse interventions rather than evidence of a single clinically uniform probiotic or synbiotic strategy, and their applicability to broader pediatric populations with overweight or obesity should be considered with caution. Third, some studies provided dietary, exercise, or lifestyle guidance alongside probiotic or synbiotic supplementation, and the intensity and implementation of these co-interventions varied across studies, making it difficult to isolate the independent effects of probiotics or synbiotics. Therefore, the observed effects should be interpreted with caution because they cannot be attributed solely to probiotic or synbiotic supplementation, and the potential contribution of lifestyle-related co-interventions should be considered when interpreting the pooled results. Fourth, some studies had small sample sizes and insufficient reporting of randomization, allocation concealment, blinding, deviations from intended interventions, or prespecified statistical analysis plans, increasing the risk of bias. These limitations may have influenced the certainty of the pooled estimates by introducing potential selection bias, performance bias, or reporting bias, thereby reducing confidence in the observed effects. Finally, subgroup analyses were only performed according to intervention type because the limited number of studies and insufficient reporting of relevant characteristics prevented reliable subgroup analyses based on probiotic strain, single- versus multi-strain preparations, dosage, intervention duration, obesity phenotype, baseline metabolic characteristics, or concomitant lifestyle interventions. Therefore, these subgroup findings should be interpreted cautiously, and future studies are needed to better explore potential sources of heterogeneity.

Future studies should also assess treatment adherence and compliance, as adherence may influence the effectiveness and interpretation of probiotic or synbiotic interventions. In addition, standardized cointervention protocols, including dietary and physical activity recommendations, should be considered to reduce variability across studies and improve comparability of treatment effects. Future trials should clearly report strain names, viable doses, supplementation duration, administration form, and product quality control, and should standardize or carefully document background dietary, exercise, and lifestyle interventions. Future research should also prioritize clinically interpretable outcomes, such as BMI z-score, body fat percentage, waist circumference, insulin sensitivity indicators, and long-term follow-up outcomes. Mechanistic indicators, including gut microbiota sequencing, short-chain fatty acids, inflammatory markers, and bile acid metabolism, should be incorporated when possible to explore whether specific strains, populations, or metabolic profiles may influence treatment responses [36,44].

## 5. Conclusions

This systematic review and meta-analysis of 16 randomized controlled trials suggests that probiotic or synbiotic supplementation may be associated with a small reduction in BMI in children and adolescents with overweight or obesity. However, current evidence does not show significant improvements in BMI z-score, waist circumference, body fat percentage, fasting glucose, fasting insulin, or HOMA-IR. Therefore, the clinical relevance of the observed BMI reduction remains uncertain, particularly because it was not accompanied by improvements in BMI z-score or other anthropometric outcomes. Furthermore, the observed reduction in BMI was supported by low-certainty evidence, which further limits confidence in its clinical implications. Exploratory subgroup analyses suggested a potential difference between probiotic and synbiotic supplementation for fasting glucose; however, this finding should be interpreted cautiously because of the limited number of studies. Further high-quality randomized controlled trials are warranted.

## Figures and Tables

**Table 1:** PICOS criteria for study eligibility.

PICOS	Eligibility Criteria
P: Population	Children and adolescents with overweight or obesity.
I: Intervention	Probiotic or synbiotic supplementation, used either alone or in combination with dietary, exercise, or lifestyle guidance.
C: Comparator	Placebo, usual care, no intervention, or a control intervention without probiotic or synbiotic supplementation.
O: Outcomes	At least one of the following outcomes: BMI z-score, BMI, waist circumference, body fat percentage, fasting glucose, fasting insulin, or HOMA-IR.
S: Study design	Randomized controlled trials

**Table 2:** Summary of findings and certainty of evidence for main outcomes.

Outcome	No. of Studies	Participants (Intervention/Control)	Pooled Effect (MD, 95% CI)	Certainty of Evidence	Downgrading Factors	Importance
BMI z-score	10	233/228	MD 0.03 BMI z-score lower (0.11 lower to 0.05 higher)	Moderate	Imprecision	Critical
BMI	9	248/242	MD 0.91 kg/m^2^ lower (1.81 lower to 0.01 lower)	Low	Inconsistency; Imprecision	Critical
Waist circumference	10	281/272	MD 0.60 cm higher (1.57 lower to 2.77 higher)	Low	Imprecision	Critical
Body fat percentage	4	97/94	MD 0.29 percentage points lower (1.64 lower to 1.06 higher)	Low	Imprecision	Critical
Fasting glucose	8	209/206	MD 0.40 mg/dL lower (2.81 lower to 2.00 higher)	Moderate	Imprecision	Important
Fasting insulin	6	137/138	MD 0.46 μIU/mL higher (1.48 lower to 2.40 higher)	Moderate	Imprecision	Important
HOMA-IR	7	172/173	MD 0.14 lower (0.57 lower to 0.28 higher)	Moderate	Imprecision	Important

Notes: MD, mean difference; CI, confidence interval; BMI, body mass index; HOMA-IR, homeostatic model assessment of insulin resistance. The certainty of evidence was assessed using the GRADE approach. Downgrading factors are presented according to the relevant GRADE domains, including inconsistency and imprecision where applicable.

**Table 3:** Characteristics of included studies.

Study	Country	Participants; Age	Sample Size (I/C)	Baseline BMI Status/Obesity Severity	Design; Duration	Intervention	Control/Co-Intervention	Outcomes
Alisi 2014 [20]	Italy	Obese children with biopsy-proven NAFLD/NASH; ~12 y	44 (22/22)	Obesity (BMI ≈ 26–27 kg/m^2^)	Parallel, double-blind; 16 wk	MSP: VSL#3	Placebo; diet/exercise advice	BMI, BMI z-score
Chen 2022 [21]	Taiwan, China	Children with overweight/obesity; 6–18 y	53 (27/26)	Obesity (BMI ≈ 29.7 kg/m^2^)	Parallel, double-blind; 12 wk	MSP: L. salivarius AP-32, L. rhamnosus bv-77, B. animalis CP-9	Placebo; diet/exercise guidance	BMI, WC, FG, FI, HOMA-IR
Famouri 2017 [22]	Iran	Obese children/adolescents with NAFLD; ~12 y	64 (32/32)	Obesity (BMI ≈ 26.5 kg/m^2^)	Parallel, triple-blind; 12 wk	MSP capsule	Placebo; healthy lifestyle advice	BMI, WC
Gøbel 2012 [23]	Denmark	Adolescents with obesity; 12–15 y	50 (27/23)	Obesity (BMI z-score ≈ 2.6)	Parallel, double-blind; 12 wk	SSP: L. salivarius Ls-33, 10^10^ CFU/d	Placebo	BMI z-score, WC, body fat%, FG, FI, HOMA-IR
Ipar 2015 [24]	Turkey	Children with primary obesity; 5–17 y	77 (42/35)	Obesity (BMI ≈ 27 kg/m^2^)	Parallel, open-label; 4 wk	SYN + reduced-calorie diet/physical activity	Reduced-calorie diet/physical activity	BMI, WC
Charoensriwattanakul 2026 [15]	Thailand	Children with obesity; 7–18 y	57 (28/29)	Obesity (BMI z-score ≈ 3.0)	Parallel, double-blind; 12 wk	SYN: inulin + B. animalis and L. paracasei	Maltodextrin placebo; lifestyle counseling	BMI z-score, BMI, WC, FG, FI, HOMA-IR
Kilic Yildirim 2022 [25]	Turkey	Children/adolescents with exogenous obesity; 8–17 y	61 (30/31)	Obesity (BMI z-score ≈ 1.7–1.8)	Parallel, double-blind; 12 wk	Multispecies SYN + FOS	Placebo; diet/activity advice	BMI z-score, BMI, WC, FG, FI, HOMA-IR
Rodrigo 2022 [26]	Sri Lanka	Obese children with NAFLD/NASH; 5–15 y	84 (43/41)	Obesity (BMI z-score ≈ 2.2)	Parallel, double-blind; 24 wk	MSP + structured diet/physical activity	Placebo + structured diet/physical activity	BMI z-score, FG
Safavi 2013 [27]	Iran	Children/adolescents with overweight/obesity; 6–18 y	56 (29/27)	Obesity (BMI ≈ 28–30 kg/m^2^)	Parallel, triple-masked; 8 wk	SYN capsule + FOS	Matched placebo; usual diet/activity	BMI z-score, WC, FG
Sanchis-Chordà 2019 [28]	Spain	Children with obesity and insulin resistance; 10–15 y	48 (23/25)	Obesity (BMI SDS ≈ 2.6)	Parallel, blinded; 13 wk	SSP: B. pseudocatenulatum CECT 7765 + dietary advice	Capsules without probiotic + same dietary advice	BMI z-score, WC, body fat%, FG, FI, HOMA-IR
Vajro 2011 [29]	Italy	Obese children with persistent hypertransaminasemia and bright liver; 10.7 ± 2.1 y	20 (10/10)	Obesity(BMI z-score ≈ 2.2)	Parallel, double-blind pilot; 8 wk	SSP: L. rhamnosus GG, 12 billion CFU/d	Placebo	BMI z-score
Verma 2021 [30]	USA	Adolescents with severe obesity; ~15.8 y	8 completers (4/4); 15 randomized	Severe obesity (BMI ≈ 45.7 kg/m^2^)	Parallel, double-blind pilot; 12 wk	Probiotic: Visbiome^®^	Placebo	BMI, BMI z-score, FG, FI, HOMA-IR
Kianifar 2018 [31]	Iran	Overweight or obese children; 7–13 y	33 completed (17/16); 46 randomized	Obesity (BMI z-score ≈ 2.0)	Parallel, double-blind pilot; 12 wk	Weight-loss diet/physical activity + SYN capsule (Protexin)	Same diet/activity + matched placebo	BMI z-score, WC, body fat%
Atazadegan 2023 [14]	Iran	Children/adolescents with overweight/obesity; 8–18 y	60 (30/30)	Obesity (BMI ≈ 25 kg/m^2^)	Parallel, double-blind; 8 wk	SYN: L. coagulans SC-208, L. indicus HU36 + FOS	Maltodextrin placebo; usual diet/activity maintained	BMI, WC
Zhang 2024 [32]	China	Adolescents with obesity; 10–18 y	60 (30/30)	Obesity (BMI ≈ 30 kg/m^2^)	Parallel RCT; 12 wk	Bifidobacterium quadruple viable tablets + diet/exercise guidance	Diet/exercise guidance	BMI, body fat%
Liu 2023 [33]	China	Obese children with NAFLD; 6–17 y	70 in main comparison (35/35); 105 randomized	Obesity(BMI ≈ 24–25 kg/m^2^)	Three-arm parallel RCT; 12 wk	Probiotic (Bifidobacterium triple viable powder) + nutrition/lifestyle intervention (Group A)	Nutrition/lifestyle intervention; vitamin D + probiotic arm not pooled	BMI, HOMA-IR

## Data Availability

No new data were created or analyzed in this study. Data sharing is not applicable to this article.

## Supplementary Materials

The following supporting information can be downloaded at: https://www.mdpi.com/article/10.3390/nu18152539/s1, File S1: PRISMA 2020 checklist; File S2: Complete search strategies for all databases; File S3: Supplementary Tables S1–S6: Supplementary Table S1. Detailed intervention characteristics and individual study-level outcome findings of probiotic and synbiotic interventions included in the meta-analysis; Supplementary Table S2. Leave-one-study-out sensitivity analysis for BMI; Supplementary Table S3. Leave-one-study-out sensitivity analysis for BMI z-score; Supplementary Table S4. Leave-one-study-out sensitivity analysis for fasting glucose; Supplementary Table S5. Leave-one-study-out sensitivity analysis for fasting insulin; Supplementary Table S6. Leave-one-study-out sensitivity analysis for HOMA-IR. Additional study characteristics and sensitivity analyses.

## Abbreviations

The following abbreviations are used in this manuscript:

BMI	Body mass index
CI	Confidence interval
CNKI	China National Knowledge Infrastructure
CFU	Colony-forming units
FOS	Fructooligosaccharide
FG	Fasting glucose
FI	Fasting insulin
GRADE	Grading of Recommendations Assessment, Development and Evaluation
HOMA-IR	Homeostatic Model Assessment of Insulin Resistance
MD	Mean difference
NAFLD	Non-alcoholic fatty liver disease
NASH	Non-alcoholic steatohepatitis
PICOS	Population, intervention, comparator, outcomes, and study design
PRISMA	Preferred Reporting Items for Systematic Reviews and Meta-Analyses
RCT	Randomized controlled trial
RoB 2	Revised Cochrane risk-of-bias tool for randomized trials, version 2
WC	Waist circumference
