# Supplementary material for: Effects of Probiotic and Synbiotic Supplementation on Body Composition and Glucose Metabolism in Children and Adolescents with Overweight or Obesity: A Systematic Review and Meta-Analysis
Source: Nutrients. 2026 Aug 4;18(15):2539. doi: 10.3390/nu18152539 (PMC13468473; doi:10.3390/nu18152539)
Supplement: Supplementary file 1 [file nutrients-18-02539-s001.zip › Supplementary File S2. Search Strategies.pdf]

Supplementary Material S2.

Detailed Search Strategies for PubMed, Web of Science, Embase, Cochrane Library, and CNKI

PubMed(494),

(("Adolescent Obesity"[Title/Abstract] OR "Adolescent Overweight"[Title/Abstract] OR "Child Obesity"[Title/Abstract] OR "Childhood Obesity"[Title/Abstract] OR "Childhood Onset Obesity"[Title/Abstract] OR "Childhood Overweight"[Title/Abstract] OR "Obesity in Adolescence"[Title/Abstract] OR "Obesity in Childhood"[Title/Abstract] OR "Overweight"[MeSH Terms] OR "Pediatric Obesity"[MeSH Terms] OR "Pediatric Obesity"[Title/Abstract] OR "excess weight"[Title/Abstract] OR "juvenile obesity"[Title/Abstract] OR "kids obesity"[Title/Abstract] OR "obese adolescents"[Title/Abstract] OR "obese youth"[Title/Abstract] OR "overweight adolescents"[Title/Abstract] OR "overweight children"[Title/Abstract] OR "overweight kids"[Title/Abstract] OR "overweight youth"[Title/Abstract] OR "peds obesity"[Title/Abstract]) AND ("Adolescence"[Title/Abstract] OR "Adolescent"[MeSH Terms] OR "Adolescents"[Title/Abstract] OR "Child"[MeSH Terms] OR "Children"[Title/Abstract] OR "Female Adolescent"[Title/Abstract] OR "Male Adolescent"[Title/Abstract] OR "Minors"[Title/Abstract] OR "Only Child"[Title/Abstract] OR "Teen"[Title/Abstract] OR "Teenager"[Title/Abstract] OR "Teenagers"[Title/Abstract] OR "Teens"[Title/Abstract] OR "Youth"[Title/Abstract] OR "Youths"[Title/Abstract] OR "childhood"[Title/Abstract] OR "childrens"[Title/Abstract] OR "pediatric"[Title/Abstract] OR "pubertal"[Title/Abstract] OR "teenage"[Title/Abstract])) AND (("Blood Glucose"[MeSH Terms] OR "Body Weight"[MeSH Terms] OR "Lipids"[MeSH Terms] OR "Metabolism"[MeSH Terms]) AND ("Inflammation"[MeSH Terms]) AND ("Anabolism"[Title/Abstract] OR "BMI"[Title/Abstract] OR "Blood Sugar"[Title/Abstract] OR "Body Weight"[Title/Abstract] OR "Body Weights"[Title/Abstract] OR "Catabolism"[Title/Abstract] OR "Fats"[Title/Abstract] OR "Glycated Hemoglobin"[Title/Abstract] OR "Glycemic Index"[Title/Abstract] OR "HDL C"[Title/Abstract] OR "HOMA IR"[Title/Abstract] OR "HbA1c"[Title/Abstract] OR "Hyperlipidemias"[Title/Abstract] OR "Hypolipidemic Agents"[Title/Abstract] OR "LDL C"[Title/Abstract] OR "Lipid"[Title/Abstract] OR "Metabolic Concept"[Title/Abstract] OR "Metabolic Concepts"[Title/Abstract] OR "Metabolic Phenomena"[Title/Abstract] OR "Metabolic Phenomenon"[Title/Abstract] OR "Metabolic Process"[Title/Abstract] OR "Metabolic Processes"[Title/Abstract] OR "Metabolism Concept"[Title/Abstract] OR "Metabolism Concepts"[Title/Abstract] OR "Metabolism Phenomena"[Title/Abstract] OR "TC"[Title/Abstract] OR "TG"[Title/Abstract] OR "body mass index"[Title/Abstract] OR "cholesterol levels"[Title/Abstract] OR "energy expenditure"[Title/Abstract] OR "glucose"[Title/Abstract] OR "insulin"[Title/Abstract] OR "lipids"[Title/Abstract] OR "metabolic index"[Title/Abstract] OR "metabolic markers"[Title/Abstract] OR "metabolic parameter"[Title/Abstract] OR "metabolic profile"[Title/Abstract] OR "metabolic rate"[Title/Abstract] OR "total cholesterol"[Title/Abstract] OR "triglycerides"[Title/Abstract] OR "weight change"[Title/Abstract]) AND ("Acute Phase Reaction"[Title/Abstract] OR "C reactive protein"[Title/Abstract] OR "CRP"[Title/Abstract] OR "IL 6"[Title/Abstract] OR "Inflammations"[Title/Abstract] OR "Innate Immunity"[Title/Abstract] OR "Innate Inflammatory Response"[Title/Abstract] OR "Innate Inflammatory Responses"[Title/Abstract]

OR "TNF alpha"[Title/Abstract] OR "adiponectin"[Title/Abstract] OR "cytokines"[Title/Abstract] OR "inflammatory marker"[Title/Abstract] OR "inflammatory markers"[Title/Abstract] OR "inflammatory parameter"[Title/Abstract] OR "interleukin 6"[Title/Abstract] OR "leptin"[Title/Abstract] OR "tumor necrosis factor"[Title/Abstract]))

Web of Science(350)

(TS=("Overweight" OR "Pediatric Obesity" OR "Adolescent Obesity" OR "Adolescent Overweight" OR "Child Obesity" OR "Childhood Obesity" OR "Childhood Onset Obesity" OR "Childhood Overweight" OR "Obesity in Adolescence" OR "Obesity in Childhood" OR "excess weight" OR "juvenile obesity" OR "kids obesity" OR "obese adolescents" OR "obese youth" OR "overweight adolescents" OR "overweight children" OR "overweight kids" OR "overweight youth" OR "peds obesity") AND TS=("Adolescent" OR "Child" OR "Adolescence" OR "Adolescents" OR "Children" OR "Female Adolescent" OR "Male Adolescent" OR "Minors" OR "Only Child" OR "Teen" OR "Teenager" OR "Teenagers" OR "Teens" OR "Youth" OR "Youths" OR "childhood" OR "childrens" OR "pediatric" OR "pubertal" OR "teenage")) AND (TS=("Blood Glucose" OR "Body Weight" OR "Lipids" OR "Metabolism") AND TS=("Inflammation") AND TS=("Anabolism" OR "BMI" OR "Blood Sugar" OR "Body Weight" OR "Body Weights" OR "Catabolism" OR "Fats" OR "Glycated Hemoglobin" OR "Glycemic Index" OR "HDL C" OR "HOMA IR" OR "HbA1c" OR "Hyperlipidemias" OR "Hypolipidemic Agents" OR "LDL C" OR "Lipid" OR "Metabolic Concept" OR "Metabolic Concepts" OR "Metabolic Phenomena" OR "Metabolic Phenomenon" OR "Metabolic Process" OR "Metabolic Processes" OR "Metabolism Concept" OR "Metabolism Concepts" OR "Metabolism Phenomena" OR "TC" OR "TG" OR "body mass index" OR "cholesterol levels" OR "energy expenditure" OR "glucose" OR "insulin" OR "lipids" OR "metabolic index" OR "metabolic markers" OR "metabolic parameter" OR "metabolic profile" OR "metabolic rate" OR "total cholesterol" OR "triglycerides" OR "weight change") AND TS=("Acute Phase Reaction" OR "C reactive protein" OR "CRP" OR "IL 6" OR "Inflammations" OR "Innate Immunity" OR "Innate Inflammatory Response" OR "Innate Inflammatory Responses" OR "TNF alpha" OR "adiponectin" OR "cytokines" OR "inflammatory marker" OR "inflammatory markers" OR "inflammatory parameter" OR "interleukin 6" OR "leptin" OR "tumor necrosis factor"))

Embase(131)

((('Overweight'/exp OR 'Pediatric Obesity'/exp) OR ('Adolescent Obesity':ti,ab OR 'Adolescent Overweight':ti,ab OR 'Child Obesity':ti,ab OR 'Childhood Obesity':ti,ab OR 'Childhood Onset Obesity':ti,ab OR 'Childhood Overweight':ti,ab OR 'Obesity in Adolescence':ti,ab OR 'Obesity in Childhood':ti,ab OR 'Pediatric Obesity':ti,ab OR 'excess weight':ti,ab OR 'juvenile obesity':ti,ab OR 'kids obesity':ti,ab OR 'obese adolescents':ti,ab OR 'obese youth':ti,ab OR 'overweight adolescents':ti,ab OR 'overweight children':ti,ab OR 'overweight kids':ti,ab OR 'overweight youth':ti,ab OR 'peds obesity':ti,ab)) AND (('Adolescent'/exp OR 'Child'/exp) OR ('Adolescence':ti,ab OR 'Adolescents':ti,ab OR 'Children':ti,ab OR 'Female Adolescent':ti,ab OR 'Male Adolescent':ti,ab OR 'Minors':ti,ab OR 'Only Child':ti,ab OR 'Teen':ti,ab OR 'Teenager':ti,ab OR 'Teenagers':ti,ab OR 'Teens':ti,ab OR 'Youth':ti,ab OR 'Youths':ti,ab OR 'childhood':ti,ab OR 'childrens':ti,ab OR 'pediatric':ti,ab OR 'pubertal':ti,ab OR 'teenage':ti,ab))) AND (('Blood Glucose'/exp OR 'Body Weight'/exp OR 'Lipids'/exp OR 'Metabolism'/exp) AND

'Inflammation'/exp AND ('Anabolism':ti,ab OR 'BMI':ti,ab OR 'Blood Sugar':ti,ab OR 'Body Weight':ti,ab OR 'Body Weights':ti,ab OR 'Catabolism':ti,ab OR 'Fats':ti,ab OR 'Glycated Hemoglobin':ti,ab OR 'Glycemic Index':ti,ab OR 'HDL C':ti,ab OR 'HOMA IR':ti,ab OR 'HbA1c':ti,ab OR 'Hyperlipidemias':ti,ab OR 'Hypolipidemic Agents':ti,ab OR 'LDL C':ti,ab OR 'Lipid':ti,ab OR 'Metabolic Concept':ti,ab OR 'Metabolic Concepts':ti,ab OR 'Metabolic Phenomena':ti,ab OR 'Metabolic Phenomenon':ti,ab OR 'Metabolic Process':ti,ab OR 'Metabolic Processes':ti,ab OR 'Metabolism Concept':ti,ab OR 'Metabolism Concepts':ti,ab OR 'Metabolism Phenomena':ti,ab OR 'TC':ti,ab OR 'TG':ti,ab OR 'body mass index':ti,ab OR 'cholesterol levels':ti,ab OR 'energy expenditure':ti,ab OR 'glucose':ti,ab OR 'insulin':ti,ab OR 'lipids':ti,ab OR 'metabolic index':ti,ab OR 'metabolic markers':ti,ab OR 'metabolic parameter':ti,ab OR 'metabolic profile':ti,ab OR 'metabolic rate':ti,ab OR 'total cholesterol':ti,ab OR 'triglycerides':ti,ab OR 'weight change':ti,ab) AND ('Acute Phase Reaction':ti,ab OR 'C reactive protein':ti,ab OR 'CRP':ti,ab OR 'IL 6':ti,ab OR 'Inflammations':ti,ab OR 'Innate Immunity':ti,ab OR 'Innate Inflammatory Response':ti,ab OR 'Innate Inflammatory Responses':ti,ab OR 'TNF alpha':ti,ab OR 'adiponectin':ti,ab OR 'cytokines':ti,ab OR 'inflammatory marker':ti,ab OR 'inflammatory markers':ti,ab OR 'inflammatory parameter':ti,ab OR 'interleukin 6':ti,ab OR 'leptin':ti,ab OR 'tumor necrosis factor':ti,ab))

Cochrane(146)

(MeSH descriptor: [Pediatric Obesity] explode all trees) OR (MeSH descriptor: [Overweight] explode all trees) OR ("child obesity" OR "childhood obesity" OR "childhood onset obesity" OR "pediatric obesity" OR "juvenile obesity" OR "adolescent obesity" OR "obesity in childhood" OR "obesity in adolescence" OR "child overweight" OR "childhood overweight" OR "adolescent overweight" OR "overweight children" OR "overweight adolescents" OR "overweight youth" OR "overweight kids" OR "obese youth" OR "obese adolescents" OR "kids obesity" OR "excess weight" OR "peds obesity"):ti,ab,kw

(MeSH descriptor: [Child] explode all trees)OR(MeSH descriptor: [Adolescent] explode all trees)OR(child*OR adolescen*OR pediatric*
OR paediatric*OR youth*OR teen*OR teenage*OR pubertal*OR minor*OR schoolchild*):ti,ab,kw
adolescen*

(MeSH descriptor: [Body Weight] explode all trees)OR(MeSH descriptor: [Blood Glucose] explode all trees)OR(MeSH descriptor: [Lipids] explode all trees)OR(MeSH descriptor: [Metabolism] explode all trees)OR(BMI OR "body mass index"OR weightOR "weight change"OR glucosOR insulinOR "blood sugar"OR HbA1cOR "glycated hemoglobin"OR HOMA-IROR "HOMA IR"OR lipids
OR triglyceridesOR TGOR cholesterolOR "total cholesterol"OR HDLOR "HDL-C"OR LDLOR "LDL-C"OR metabolismOR "metabolic profile"OR "metabolic marker*"OR "metabolic parameter*"OR "metabolic rate"OR "energy expenditure"):ti,ab,kw

(MeSH descriptor: [Inflammation] explode all trees)OR(CRPOR "C-reactive protein"OR inflammationOR inflammatoryOR cytokine*OR leptinOR adiponectinOR "interleukin-6"OR IL-6OR "tumor necrosis factor"OR TNF-alphaOR "TNF alpha"OR "inflammatory marker*"OR "inflammatory parameter*"

#1 AND #2 AND #3 AND #4

CNKI(34)

（益生菌 OR 合生元 OR 双歧杆菌 OR 乳杆菌 OR 乳酸菌）AND（儿童肥胖 OR 青少年肥胖 OR 肥胖儿童 OR 肥胖青少年 OR 非酒精性脂肪肝 OR 非酒精性脂肪性肝病）AND（随机 OR 对照 OR 安慰剂 OR 临床试验）
