# Supplementary material for: Effects of Probiotic and Synbiotic Supplementation on Body Composition and Glucose Metabolism in Children and Adolescents with Overweight or Obesity: A Systematic Review and Meta-Analysis
Source: Nutrients. 2026 Aug 4;18(15):2539. doi: 10.3390/nu18152539 (PMC13468473; doi:10.3390/nu18152539)
Supplement: Supplementary file 1 [file nutrients-18-02539-s001.zip › Supplementary File S3 Tables S1–S6 Additional study characteristics and sensitivity analyses..pdf]

Supplementary Table S1. Detailed intervention characteristics and individual study-level outcome findings of probiotic and synbiotic interventions included in the meta-analysis

Abbreviations: BMI, body mass index; WC, waist circumference; FG, fasting glucose; FI, fasting insulin; HOMA-IR, homeostatic model assessment of insulin resistance; CFU, colony-forming units.

Study	Intervention type	Strain/product	Dose	Duration	Population	Individual study-level outcome findings
Alisi 2014[20]	Probiotic	VSL#3 multi-strain probiotic	9×10^{11} CFU/day	16 weeks	Obesity; ~12 y	No significant between-group differences in BMI or BMI z-score
Chen 2022[21]	Probiotic	L. salivarius AP-32, L. rhamnosus bv-77, B. animalis CP-9	1×10^{10} CFU/day	12 weeks	Overweight/obesity; 6–18 y	No significant between-group effects on BMI, WC, FG, FI, or HOMA-IR
Famouri 2017[22]	Probiotic	Multi-strain probiotic (L. acidophilus, B. lactis, B. bifidum, L. rhamnosus)	1.3×10^{10} CFU/day	12 weeks	Obesity; ~12 y	Reduced WC; no significant between-group differences in BMI or BMI z-score
Gøbel 2012[23]	Probiotic	Lactobacillus salivarius Ls-33	1×10^{10} CFU/day	12 weeks	Obesity; 12–15 y	No significant effects on BMI z-score, WC, body fat %, FG, FI, or HOMA-IR
Ipar 2015[24]	Synbiotic	Multi-strain synbiotic formulation plus prebiotic	Not clearly specified	4 weeks	Obesity; 5–17 y	Reported changes in anthropometric outcomes

						including BMI-related outcomes
Charoensriwattanakul 2026[25]	Synbiotic	Inulin plus B. animalis and L. paracasei	Not clearly specified	12 weeks	Obesity; 7–18 y	No significant between-group effects on BMI, BMI z-score, WC, FG, FI, or HOMA-IR
Rodrigo 2022[26]	Probiotic	Bio-Kult® 14-strain probiotic preparation	Dose according to age group as reported in the original article	24 weeks	Obesity; 5–15 y	No significant between-group effects on BMI-related outcomes or FG
Kilic Yildirim 2022[27]	Synbiotic	Multi-species synbiotic formulation containing probiotic strains plus prebiotic components	Dose as reported in the original article	12 weeks	Obesity; 8–17 y	Reported changes in BMI, BMI z-score, and WC; no significant effects on FG, FI, or HOMA-IR
Safavi 2013[28]	Synbiotic	Multi-strain synbiotic containing Lactobacillus and Bifidobacterium strains plus FOS	Not clearly specified	8 weeks	Overweight/obesity; 6–18 y	No significant between-group effects on BMI z-score, WC, or FG
Sanchis-Chordà 2019[29]	Probiotic	Bifidobacterium pseudocatenulatum CECT 7765	10^{10} CFU/day	13 weeks	Obesity; 10–15 y	No significant effects on anthropometric or glucose metabolism outcomes
Vajro 2011[30]	Probiotic	Lactobacillus	12	8	Obesity; 10–11	No

		rhamnosus GG	billion CFU/day	weeks	y	significant effect on BMI z-score
Verma 2021[31]	Probiotic	Visbiome® multi-strain probiotic	Not clearly specified	12 weeks	Severe obesity; ~16 y	No significant between-group differences in BMI-related and metabolic outcomes
Kianifar 201[32]	Synbiotic	Protexin synbiotic capsule	Not clearly specified	12 weeks	Overweight/obesity; 7–13 y	Reported changes in anthropometric indices; included in BMI-related analyses
Atazadegan 2023[33]	Synbiotic	L. coagulans SC-208 plus L. indicus HU36 and FOS	Not clearly specified	8 weeks	Overweight/obesity; 8–18 y	Reported changes in anthropometric and body composition outcomes
Liu 2023[34]	Probiotic	Bifidobacterium triple viable powder	Not clearly specified	12 weeks	Obesity; 6–17 y	Probiotic group compared with lifestyle intervention control; reported changes in BMI and HOMA-IR
Zhang 2024[35]	Probiotic	Bifidobacterium quadruple viable tablets	Not clearly specified	12 weeks	Obesity; 10–18 y	Reported reductions in BMI and changes in body composition-related parameters

Supplementary Table S2. Leave-one-study-out sensitivity analysis for BMI

Excluded study	MD (95% CI)	P value	Effect stability
None (overall analysis)	−0.91 (−1.81, −0.01)	P < 0.05	—
Alisi 2014	−0.92 (−1.92, 0.08)	>0.05	Stable
Atazadegan 2023	−0.91 (−1.88, 0.05)	>0.05	Stable
Charoensriwattanakul 2026	−1.09 (−2.00, −0.18)	<0.05	Stable
Chen 2022	−0.91 (−1.88, 0.05)	>0.05	Stable
Ipar 2015	−0.77 (−1.73, 0.19)	>0.05	Stable
Kilic Yildirim 2022	−0.88 (−2.07, 0.32)	>0.05	Stable
Liu 2023	−0.62 (−1.55, 0.32)	>0.05	Stable
Verma 2021	−0.86 (−1.78, 0.06)	>0.05	Stable
Zhang 2024	−0.74 (−1.77, 0.29)	>0.05	Stable

Note:Leave-one-study-out sensitivity analyses were performed by sequentially removing each individual study. The pooled BMI estimates remained consistent in direction and magnitude after exclusion of individual studies, suggesting that no single study substantially influenced the overall effect estimate.

Supplementary Table S3. Leave-one-study-out sensitivity analysis for BMI z-score

Excluded study	MD (95% CI)	P value	Effect stability
None (overall analysis)	−0.03 (−0.11, 0.05)	>0.05	—
Alisi 2014	0.00 (−0.09, 0.10)	>0.05	Stable
Charoensriwattanakul 2026	−0.02 (−0.11, 0.06)	>0.05	Stable
Gøbel 2012	−0.01 (−0.09, 0.07)	>0.05	Stable
Kianifar 2018	−0.06 (−0.13, 0.02)	>0.05	Stable
Kilic Yildirim 2022	−0.02 (−0.11, 0.07)	>0.05	Stable
Rodrigo 2022	−0.02 (−0.11, 0.07)	>0.05	Stable
Safavi 2013	−0.03 (−0.12, 0.06)	>0.05	Stable
Sanchis-Chordà 2019	−0.02 (−0.11, 0.07)	>0.05	Stable
Vajro 2011	−0.02 (−0.10, 0.06)	>0.05	Stable
Verma 2021	−0.02 (−0.11, 0.06)	>0.05	Stable

Note: Leave-one-study-out sensitivity analyses were performed by sequentially removing each included study. The pooled estimates remained consistent in direction, magnitude, and statistical

significance after exclusion of individual studies, indicating that no single study substantially influenced the overall effect estimate.

Supplementary Table S4. Leave-one-study-out sensitivity analysis for fasting glucose

Excluded study	MD (95% CI)	P value	Effect stability
None (overall analysis)	−0.40 (−2.81, 2.00)	>0.05	—
Charoensriwattanakul 2026	−0.31 (−3.07, 2.46)	>0.05	Stable
Chen 2022	0.02 (−2.53, 2.58)	>0.05	Stable
Gøbel 2012	−0.85 (−3.58, 1.88)	>0.05	Stable
Kilic Yildirim 2022	0.08 (−2.43, 2.59)	>0.05	Stable
Rodrigo 2022	−0.52 (−3.20, 2.16)	>0.05	Stable
Safavi 2013	0.24 (−2.05, 2.54)	>0.05	Stable
Sanchis-Chordà 2019	−0.86 (−3.51, 1.80)	>0.05	Stable
Verma 2021	−0.52 (−3.20, 2.16)	>0.05	Stable

Note:Leave-one-study-out sensitivity analyses were performed by sequentially removing each individual study. The pooled fasting glucose estimates remained consistent in direction, magnitude, and statistical significance after exclusion of individual studies, indicating that no single study substantially influenced the pooled effect estimate.

Supplementary Table S5. Leave-one-study-out sensitivity analysis for fasting insulin

Excluded study	MD (95% CI)	P value	Effect stability
None (overall analysis)	0.46 (−1.48, 2.40)	>0.05	—
Charoensriwattanakul 2026	0.25 (−1.85, 2.34)	>0.05	Stable
Chen 2022	0.23 (−2.07, 2.52)	>0.05	Stable
Gøbel 2012	0.47 (−1.75, 2.70)	>0.05	Stable
Kilic Yildirim 2022	1.13 (−0.99, 3.25)	>0.05	Stable
Sanchis-Chordà 2019	0.46 (−1.49, 2.40)	>0.05	Stable
Verma 2021	0.46 (−1.49, 2.40)	>0.05	Stable

Note:Leave-one-study-out sensitivity analyses were performed by sequentially removing each individual study. The pooled fasting insulin estimates remained consistent in direction,

magnitude, and statistical significance after exclusion of individual studies, indicating that no single study substantially influenced the pooled effect estimate.

Supplementary Table S6. Leave-one-study-out sensitivity analysis for HOMA-IR

Excluded study	MD (95% CI)	P value	Effect stability
None (overall analysis)	−0.14 (−0.57, 0.28)	>0.05	—
Charoensriwattanakul 2026	-0.19 (-0.66, 0.28)	>0.05	Stable
Chen 2022	-0.17 (-0.67, 0.33)	>0.05	Stable
Gøbel 2012	-0.17 (-0.68, 0.34)	>0.05	Stable
Kilic Yildirim 2022	0.12 (-0.31, 0.56)	>0.05	Stable
Liu 2023	-0.07 (-0.55, 0.42)	>0.05	Stable
Sanchis-Chordà 2019	-0.12 (-0.58, 0.33)	>0.05	Stable
Verma 2021	-0.07 (-0.55, 0.42)	>0.05	Stable

Note:Leave-one-study-out sensitivity analyses were performed by sequentially removing each individual study. The pooled HOMA-IR estimates remained consistent in direction, magnitude, and statistical significance after exclusion of individual studies, indicating that no single study substantially influenced the pooled effect estimate.
